# Supplementary material for: Association between Visceral Adipose Tissue Metabolism and Cerebral Glucose Metabolism in Patients with Cognitive Impairment
Source: Int J Mol Sci. 2024 Jul 8;25(13):7479. doi: 10.3390/ijms25137479 (PMC11242271; doi:10.3390/ijms25137479)
Supplement: Supplementary file 1 [file ijms-25-07479-s001.zip › Supplementary Table S1, S3, S4.pdf]

Supplementary Table S1. Cognitive function across total cohort, cognitively unimpaired, mild cognitive Impairment, and dementia subjects.

Cognitive Domain	Neuropsychological Variable	Total (n = 54)	CU (n =18)	MCI (n = 14)	Dementia (n = 22)	*p*
Attention	Digit span forward (SD)	6.0(1.7)	7.4(1.3)	6.1(1.5)	4.6(1.0)	<0.001*
Working Memory	Digit span backward (SD)	3.7(1.5)	4.7(1.3)	4.0(1.0)	2.7(1.1)	<0.001**
Language Ability	K-BNT (SD)	42.4(13.5)	52.0(4.0)	47.8(10.1)	31.1(12.2)	<0.001**
Visuospatial Ability	RCFT copy (SD)	29.7(6.3)	32.9(2.5)	31.8(3.6)	24.7(7.7)	<0.001**
Memory	SVLT immediate recall (SD)	19.4(6.2)	23.8(3.3)	20.8(6.5)	15.1(4.9)	<0.001**
Memory	SVLT delayed recall (SD)	5.4(3.6)	8.2(1.6)	6.1(3.8)	2.6(2.6)	<0.001**
Memory	SVLT recognition (SD)	20.0(2.8)	22.6(1.3)	20.6(2.1)	17.7(1.9)	<0.001*
Memory	RCFT immediate recall (SD)	11.4(6.9)	15.0(6.7)	13.0(6.2)	6.2(4.2)	<0.001**
Memory	RCFT delayed recall (SD)	10.6(7.4)	15.1(6.6)	12.7(5.3)	4.2(4.3)	<0.001**
Verbal Fluency	COWAT animal (SD)	13.8(5.5)	17.9(5.1)	14.6(4.8)	9.9(2.9)	<0.001**
Verbal Fluency	COWAT supermarket (SD)	15.6(7.3)	23.7(6.8)	17.3(5.2)	11.0(4.6)	<0.001*
Verbal Fluency	COWAT phonemic (SD)	20.0(13.7)	29.8(15.3)	24.1(12.6)	12.3(8.7)	0.001**
Executive Function	Stroop colour reading (SD)	79.2(28.4)	99.7(17.6)	80.6(15.0)	53.6(25.7)	<0.001**
Global Cognition	K-MMSE score (SD)	24.6(5.3)	28.9(1.2)	25.9(2.9)	20.2(5.2)	<0.001**

CU, cognitively unimpaired; MCI, mild cognitive impairment; SD, standard deviation; K-BNT, Korean-Boston Naming Test; RCFT, Rey Complex Figure Test; SVLT, Seoul Verbal Learning Test; COWAT, Controlled Oral Word Association Test; K-MMSE, Korean-Mini-Mental State Examination. *CU > MCI > Dementia in post hoc analysis; **CU and MCI > Dementia in post hoc analysis.

Supplementary Table S3. Comparison of cerebral glucose metabolism between low and high VAT metabolism groups.

Region	Side	Total (n = 54)	Low VAT Metabolism Group (n = 27)	High VAT Metabolism Group (n = 27)	*p*
AD signature		0.95 (0.05)	0.96 (0.05)	0.93 (0.05)	0.016
Central	Left	1.01 (0.03)	1.01 (0.02)	1.01 (0.03)	0.403
	Right	1.00 (0.03)	0.99 (0.03)	1.01 (0.03)	0.068
Lateral frontal	Left	0.97 (0.08)	0.98 (0.06)	0.95 (0.09)	0.039
	Right	0.96 (0.09)	0.97 (0.06)	0.94 (0.10)	0.088
Medial frontal	Left	0.93 (0.08)	0.93 (0.07)	0.93 (0.09)	0.447
	Right	0.93 (0.07)	0.93 (0.07)	0.93 (0.06)	0.469
Orbital frontal	Left	0.93 (0.09)	0.94 (0.08)	0.91 (0.11)	0.173
	Right	0.92 (0.09)	0.94 (0.08)	0.90 (0.10)	0.052
Insula	Left	0.97 (0.06)	1.00 (0.04)	0.95 (0.07)	0.004
	Right	0.98 (0.07)	1.01 (0.06)	0.94 (0.07)	<0.001
Lateral temporal	Left	1.04 (0.08)	1.04 (0.09)	1.03 (0.07)	0.238
	Right	1.02 (0.09)	1.04 (0.08)	1.00 (0.09)	0.051
Lateral parietal	Left	0.98 (0.10)	0.99 (0.12)	0.97 (0.06)	0.252
	Right	0.92 (0.09)	0.93 (0.09)	0.91 (0.08)	0.292
Medial parietal	Left	1.07 (0.09)	1.07 (0.12)	1.07 (0.06)	0.494
	Right	1.06 (0.09)	1.06 (0.10)	1.05 (0.07)	0.335
Lateral occipital	Left	0.96 (0.11)	0.95 (0.14)	0.97 (0.08)	0.341
	Right	0.95 (0.13)	0.96 (0.14)	0.94 (0.12)	0.244
Medial occipital	Left	1.02 (0.08)	1.03 (0.09)	1.02 (0.07)	0.342
	Right	1.03 (0.06)	1.03 (0.07)	1.02 (0.06)	0.279
Limbic	Left	0.87 (0.06)	0.88 (0.06)	0.86 (0.06)	0.095
	Right	0.80 (0.06)	0.81 (0.06)	0.79 (0.07)	0.150

All values are means (SD).

Supplementary Table S4. Association between visceral adipose tissue SUVmax and cerebral glucose metabolism in the overall cohort.

Regions	Univariable Model		Multivariable Model		
	r	*p*	Adjusted R^2	Standardized β*	*p*
AD signature	-0.340	0.012	0.226	-0.252	0.047
Left central	0.010	0.945			-
Right central	0.206	0.136			
Left lateral frontal cortex	-0.295	0.030	0.118	-0.223	0.093
Right lateral frontal cortex	-0.194	0.159			-
Left medial frontal cortex	0.015	0.916			-
Right medial frontal cortex	0.087	0.534			-
Left orbital frontal cortex	-0.087	0.530			-
Right orbital frontal cortex	-0.196	0.156			-
Left insula	-0.368	0.006	0.180	-0.304	0.021
Right insula	-0.436	0.001	0.282	-0.353	0.004
Left lateral temporal cortex	-0.159	0.251			-
Right lateral temporal cortex	-0.242	0.077			-
Left lateral parietal cortex	-0.184	0.182			-
Right lateral parietal cortex	-0.127	0.360			-
Left medial parietal cortex	0.009	0.951			-
Right medial parietal cortex	-0.066	0.636			-
Left lateral occipital cortex	0.101	0.468			-
Right lateral occipital cortex	0.033	0.811			-
Left medial occipital cortex	0.024	0.861			-
Right medial occipital cortex	0.030	0.827			-
Left limbic	-0.215	0.119			-
Right limbic	-0.198	0.152			-

*Values represent the standardized linear regression coefficients (β) of the correlation between the visceral adipose tissue's maximum standardized uptake value (SUV_{max}) and cerebral glucose metabolism, after adjusting for age, sex, and white matter hyperintensity volume.
