# Supplementary material for: Association between Visceral Adipose Tissue Metabolism and Cerebral Glucose Metabolism in Patients with Cognitive Impairment
Source: Int J Mol Sci. 2024 Jul 8;25(13):7479. doi: 10.3390/ijms25137479 (PMC11242271; doi:10.3390/ijms25137479)
Supplement: Supplementary file 1 [file ijms-25-07479-s001.zip › Supplementary Table S2.pdf]

Supplementary Table S2. Association between clinical variables and neuropsychological variables.

Characteristics		Digit span forward	Digit span backward	K-BNT	RCFT copy	SVLT immediate recall	SVLT delayed recall	SVLT recognition	RCFT immediate recall	RCFT delayed recall	COWAT animal	COWAT supermarket	COWAT phonemic	Stroop colour reading	K-MMSE score
Age	r	-0.370	-0.301	-0.506	-0.035	-0.400	-0.371	-0.307	-0.371	-0.421	-0.321	-0.293	-0.276	-0.242	-0.319
	p	0.006	0.029	<0.001	0.817	0.003	0.006	0.027	0.013	0.004	0.020	0.063	0.084	0.118	0.019
Sex	r	0.216	0.201	0.351	-0.045	0.076	0.075	0.183	-0.007	-0.059	0.362	0.212	0.419	0.081	0.115
	p	0.117	0.149	0.012	0.763	0.590	0.592	0.194	0.965	0.703	0.008	0.183	0.007	0.605	0.407
Body mass index	r	0.112	0.011	0.255	-0.048	0.135	0.075	0.213	0.251	0.254	0.145	-0.101	0.011	-0.040	0.011
	p	0.421	0.939	0.071	0.751	0.334	0.595	0.129	0.100	0.096	0.306	0.531	0.949	0.799	0.939
Education	r	0.635	0.653	0.639	0.650	0.695	0.590	0.469	0.362	0.424	0.585	0.590	0.716	0.584	0.729
	p	<0.001	<0.001	<0.001	<0.001	<0.001	<0.001	<0.001	0.016	0.004	<0.001	<0.001	<0.001	<0.001	<0.001
Diabetes	r	0.081	0.163	0.230	0.001	-0.117	-0.074	0.119	0.200	0.236	0.096	0.075	0.061	0.056	-0.041
	p	0.558	0.245	0.104	0.994	0.405	0.600	0.401	0.194	0.122	0.500	0.643	0.710	0.720	0.770
Hypertension	r	0.314	0.339	0.334	0.111	0.134	0.184	0.205	0.195	0.321	0.232	0.196	0.138	0.244	0.230
	p	0.021	0.013	0.017	0.459	0.340	0.188	0.146	0.205	0.033	0.099	0.219	0.397	0.114	0.095
Cardiovascular disease	r	0.026	0.086	-0.016	-0.041	-0.040	0.016	0.097	-0.030	-0.012	0.183	0.115	0.027	-0.047	-0.105
	p	0.849	0.540	0.911	0.784	0.774	0.909	0.494	0.847	0.939	0.195	0.474	0.871	0.764	0.452

Hyperlipidemia	r	0.105	0.039	0.080	0.259	0.082	0.059	0.177	0.122	0.087	0.086	-0.025	0.007	0.077	0.033
	p	0.449	0.782	0.576	0.079	0.561	0.673	0.209	0.429	0.574	0.545	0.879	0.965	0.626	0.812
AD signature region SUVR	r	0.355	0.275	0.471	0.235	0.402	0.390	0.386	0.448	0.456	0.243	0.253	0.231	0.424	0.429
	p	0.008	0.047	<0.001	0.112	0.003	0.004	0.005	0.002	0.002	0.083	0.111	0.151	0.005	0.001
WMH volume	r	-0.356	-0.246	-0.620	-0.332	-0.463	-0.479	-0.449	-0.393	-0.465	-0.452	-0.404	-0.389	-0.463	-0.432
	p	0.008	0.076	<0.001	0.023	<0.001	<0.001	0.001	0.008	0.001	0.001	0.009	0.013	0.002	0.001
VAT SUVmax	r	-0.140	-0.182	-0.297	-0.098	-0.122	-0.195	-0.212	-0.302	-0.234	-0.137	-0.396	-0.300	-0.273	-0.232
	p	0.313	0.191	0.034	0.513	0.383	0.161	0.131	0.047	0.127	0.331	0.010	0.060	0.077	0.091

K-BNT, Korean Boston Naming Test; RCFT, Rey Complex Figure Test; SVLT, Seoul Verbal Learning Test; COWAT, Controlled Oral Word Association Test.
